# Improving office workers’ mental health and cognition: a 3-arm cluster randomized controlled trial targeting physical activity and sedentary behavior in multi-component interventions

**DOI:** 10.1186/s12889-019-6589-4

**Published:** 2019-03-05

**Authors:** Carla F. J. Nooijen, Victoria Blom, Örjan Ekblom, Maria M. Ekblom, Lena V. Kallings

**Affiliations:** 1 The Swedish School of Sport and Health Sciences (GIH), Stockholm, Sweden; 2 The Department of Public Health Sciences, Karolinska Institutet, Stockholm, Sweden; 3 The Department of Neuroscience, Karolinska Institutet, Stockholm, Sweden; 4 Department of Public Health and Caring Sciences, Family Medicine and Preventive Medicine, Uppsala University, Uppsala, Sweden

**Keywords:** Office workers, Work place, Randomized intervention, Sedentary behavior, Physical activity, Accelerometer, Mental health, Cognition

## Abstract

**Background:**

Physically inactive and sedentary lifestyles are negatively related to both mental health and cognition. For office-workers, who spend two-thirds of their workday sitting, it is important to improve these lifestyles. The aim of this study is to assess the effectiveness of multi-component interventions, incorporating individual, environmental and organizational changes, to increase physical activity or reduce sedentary behavior among office-workers in order to improve mental health and cognition.

**Methods:**

a 3-arm, clustered randomized controlled trial (RCT) with waiting list control group amongst adult office-workers of two large Swedish companies. Cluster teams will be randomized into 6-month interventions or to a passive waiting list control group which will receive the allocated intervention with a 6-month delay. Two multicomponent interventions will be studied of which one focuses on improving physical activity and the other on reducing sedentary behavior. Both interventions include 5 sessions of motivational counselling. In the physical activity intervention persons also get access to a gym and team leaders will organize lunch walks and encourage to exercise. In the sedentary behavior intervention standing- and walking meetings will be implemented and team leaders will encourage to reduce sitting. The recruitment target is 110 office-workers per arm (330 in total). Measurements will be repeated every 6 months for a total intended duration of 24 months. Proximal main outcomes are physical activity measured with accelerometers and sedentary behavior with inclinometers. Distal outcomes are self-reported mental health and a cognition test battery. Additional outcomes will include cardiovascular fitness, body composition, sleep, self-reported physical activity and sedentary behavior, other health habits, physical health, and working mechanisms from blood samples and questionnaires.

**Discussion:**

This cluster RCT will contribute to the currently available evidence by comparing the effectiveness of multi-component interventions targeting physical activity or sedentary behavior with the end goal of improving mental health and cognition. This study is strong in its cluster randomized design, numerous objective outcome measures and long-term follow-up. The exact content of the interventions has been defined by combining theory with results from a larger research project as well as having a continuous dialogue with the involved companies.

**Trial registration:**

ISRCTN92968402.

**Electronic supplementary material:**

The online version of this article (10.1186/s12889-019-6589-4) contains supplementary material, which is available to authorized users.

## Background

Physically inactive and sedentary lifestyles are major public health problems with accumulating evidence that these lifestyles are related to increased risk of cardio metabolic health and premature mortality [1–3]. A substantial proportion of life is spent at work and ever more people have office jobs in which sitting is the default [1]. Epidemiological studies report that office workers spend at least two-thirds of their workday sitting [4, 5]. Office-workers are therefore an important target group for interventions to improve physical activity or reduce sedentary behavior.

Healthy brain functions is an umbrella term which includes mental health and cognition. To maintain healthy brain functions, the brain requires a constant supply of oxygen and other chemicals, delivered via its abundant blood vessels. Physical activity helps to circulate nutrient-rich blood efficiently throughout the body to keep the blood vessels healthy and it increases the creation of mitochondria both in our muscles and in our brain [6]. Furthermore, physical activity might enhance neurogenesis: the ability to grow new brain cells [7]. Sedentary behavior has been consistently found to be related to worse cognitive performance [8], and there is increasing evidence that physical inactivity affects several physiological mechanisms underpinning brain health with negative consequences on cognition and mental health [9–11].

These identified links of physical activity and sedentary behavior with cognition and mental health have led to our hypotheses that increasing physical activity or reducing sedentary behavior will result in better mental health and cognition. So far only few studies have looked into this. A pilot investigation among high school students studied the effects of the implementation of stand-biased school desks on brain functions, and found that continued utilization of these desks was associated with significant improvements in executive function and working memory capabilities [12]. However, another study found no-long term effects of a physical activity intervention in preadolescents on working memory or arithmetic [13]. Further controlled intervention studies clarifying this relation in population groups at risk, such as office-workers, are therefore warranted.

As it is unknown whether sedentary behavior or physical activity could have the greatest impact on mental health and cognition, it is important to compare separate interventions on physical activity and sedentary behavior to a group receiving no intervention. Physical activity interventions have shown mixed effects on improving physical activity, with some interventions succeeding in certain subgroups, resulting in an improvement of selected health outcomes, work culture, and job stress [14]. Workplace interventions aiming at reducing sedentary behavior also show inconsistent results, and it therefore remains unknown which types of interventions and delivery mode should be advised to reduce sedentary behavior [15]. For both physical activity and sedentary behaviour, there is still a lack of well conducted intervention studies among office-workers, especially with long-term follow-up of objectively measured physical activity patterns combined with brain functions outcomes [15, 16].

This study is the third subproject of a research project entitled “Physical activity and healthy brain functions”. The first subproject is a cross-sectional study among 547 office-workers to identify how different components within objectively measured physical activity patterns are associated to healthy brain functions such as mental health and cognitive function. The second subproject aims at understanding the possible mechanisms of how physical activity behaviors might promote healthy brain functions. This is achieved by investigating acute effects of three different, highly standardized but also ecologically valid types of working day physical activity patterns on brain functions. Results and data of the first parts of this project have been used in the development of the current trial; in particular for the sample size calculations and the development of the interventions [17].

## Methods

### Aim

The aim of this study is to assess the effectiveness of multi-component interventions, incorporating individual, environmental and organizational changes, to increase physical activity or reduce sedentary behavior among office-workers in order to improve mental health and cognition.

### Research questions

1. For inactive and sedentary office workers, does a multi-component intervention to promote physical activity or reduce sedentary behavior lead to more favorable physical activity and sedentary behavior?
2. Do favorable changes in physical activity or sedentary behavior result in better mental health and cognition, on the short and long term?

The primary hypothesis is that the multi-component interventions will favorably change office-workers physical activity or sedentary behavior as compared to the waiting list control group at 6 months. The secondary hypothesis is that these changes in physical activity patterns will in turn have positive longer term effects on mental health and cognition.

### Trial registration

The trial was prospectively registered as ISRCTN92968402 on 27/02/2018, recruitment started 15/03/2018. Note that only because the date of the invoice for registration of trial was set and therefore paid after recruitment registration, ISRCTN has flagged the trial as retrospectively. However an editorial note has been added to the trial registration to clarify that by scientific definition, our trial was registered prospectively, i.e. a final version of the trial registration was received and approved by ISRCTN before the start of recruitment.

### Design

**Fig. 1:**
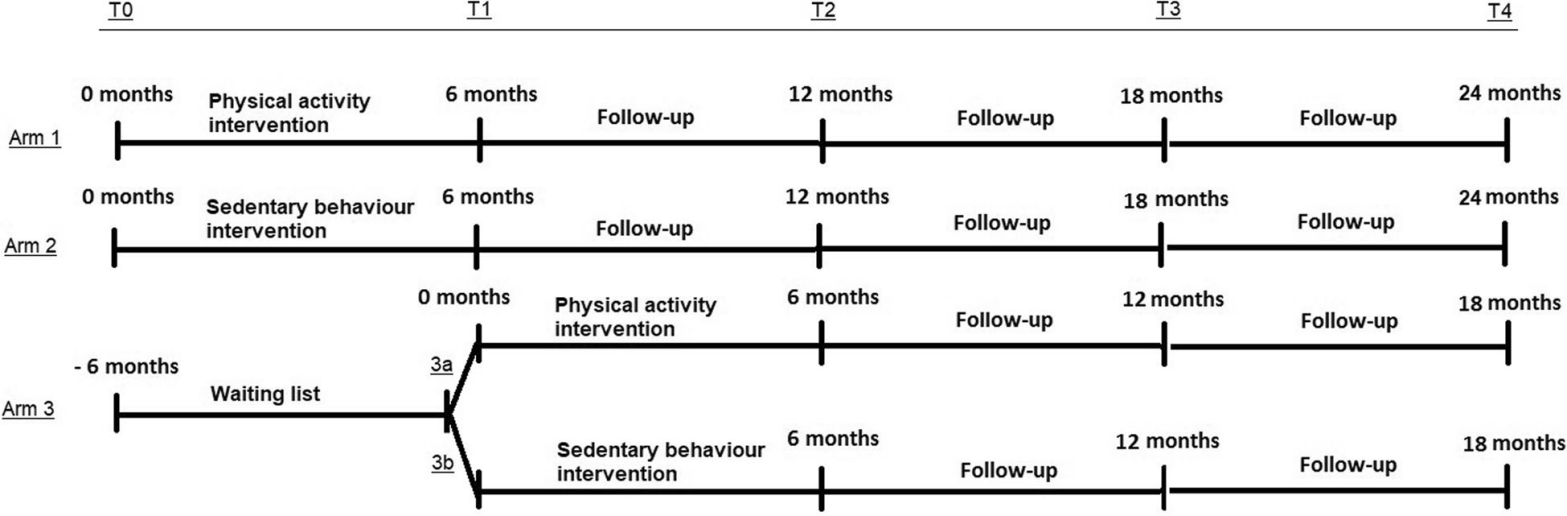
The 3 arms, with the 5 specified measurement points

The design is a 3-armed, clustered randomized controlled trial with waiting list control group, see Fig. 1. This 24-months study includes 5 assessment points. The outcomes of the randomized controlled trial are based on the first two measurement points. After that, the study continues as a cohort study with long term follow-up measurements of up to 1.5 years after the end of the intervention.

### Study population

Office workers from two Swedish companies (Intrum and ICA-gruppen) will be invited to participate in the study, with the target to include a total of 330 persons. At Intrum, persons will be recruited both working in Stockholm and in Göteborg, with respectively 168 and 265 employees. ICA-gruppen is a large company in Stockholm of around 1600 employees.

### In- and exclusion criteria

Inclusion criteria:

- Aged between 18 and 70 years of age
- Have the capability of standing and exercising

Exclusion criteria:

- Not be working for the full duration of the first study year (i.e. retirement, maternity leave)
- Very high physical activity level: more than 30 min/day in prolonged bouts (≥10 min) moderate to vigorous physical activity. This exclusion criteria will be checked by analyzing accelerometer data. Note that because subproject 1 has shown that almost all office-workers reported high levels of sedentary behavior [17], sedentary behavior will not be used as an exclusion criterion. Data collected of these persons will be excluded from the analytical sample

### Recruitment

Information meetings about the aim of the project and the research questions will be held at personnel meetings by the researchers. Furthermore, a short video will be spread on the internal webpages of the companies. Invitations to participate will be send by email and subjects can sign up online. If they thereafter chose to participate in the study, they will be assessed on eligibility.

### Randomization

In order to control contamination and to limit interaction between the different groups, randomisation will be done on a cluster level. We aim to have 24 clusters (8 clusters per arm), 10 at Intrum and 14 at ICA-gruppen. Clusters will be composed while considering: 1) having a team or line manager, 2) having regular group meetings, 3) limited regular meetings with other teams. Block randomisation and assignment will be performed using a computer-generated random number list. Groups will be randomly allocated (1:1) with stratification for company and cluster size (large vs small). In order to implement the interventions in an order consistent with logistical capacity matched randomization will be used in case needed. Randomization will be performed after finishing first data collection and participants will be notified by email. Randomization and allocation will be performed by researchers not involved in data collection. Research assistants involved in data collection and processing will be blinded for group allocation.

### Interventions

Two multicomponent interventions of 6 months will be studied. The interventions are based on the ecological framework suggesting that behavior can be influenced on multiple levels including individual, social, organizational, environmental and policy [18]. In an ecological model proposed for sedentary behavior it has been further emphasized that in order to influence behavior, strategies should target multiple levels [19]. The taxonomy of behavior change techniques was used to translate theoretical components into intervention strategies, as well as to extract effective intervention components from other behavior change techniques and are descripted in Additional file 1 [20]. The exact content of the interventions has been defined by combining this framework with results from the first and second subproject of the research project [17], as well as having a continuous dialogue with both the involved companies about the intervention in all development stages.

When performing interventions to increase physical activity or reduce sedentary behavior in one context, there is a risk that people compensate by modifying their behavior in other contexts [21]. Therefore, the interventions will focus on both work- and leisure time.

The physical activity intervention aims to promote physical activity of moderate to vigorous intensity and includes:

i. Individual: motivational counselling towards improving their time spent in moderate to vigorous physical activity, based on cognitive behavioral therapy (CBT) [22]. Including feedback on their moderate to vigorous physical activity
ii. Environmental: access to a commercial gym (6 months) as well as exercise sessions and lunch walks organized by team leaders and provision of company bikes
iii. Organizational: team leaders encourage employees to be physically active during and outside working hours, including commuting to work

The sedentary behavior intervention aims to reduce sedentary behavior, including breaking up prolonged sitting and includes:

i. Individual: motivational counselling towards reducing their time in sedentary behavior and breaking up prolonged sitting, based on CBT [22]. Including individual feedback on sedentary behaviour
ii. Environmental: implementation of standing and walking meetings, initiated by team leaders. Note that companies already provided their employees with sit-stand desks, however in the first part of the larger research project we found that usage of these desks to stand is limited [17]
iii. Organizational: team leaders encourage employees to reduce sedentary behavior at work, both in meetings and while sitting behind their desk

#### Motivational counselling

This motivational counselling has a comparable design but a different focus in either intervention, respectively aiming at physical activity or sedentary behaviour. The counselling will be performed by professional health coaches from a health promotion company Itrim who will receive additional training on CBT techniques and on physical activity and sedentary behavior. These training sessions will last for two days and will be held by CBT educated psychologists and physical activity expert. All Itrim coaches have experience with behavior counselling and are used to face-to-face meetings with clients. In total there will be five sessions; three individual and two group sessions. The first individual session is scheduled to last 60 min, the other individual sessions 45 min, and group sessions 90 min. In between session one and two there will be two weeks and thereafter sessions are 4–5 weeks apart. After session three (in the middle of the active intervention period, approximate 3 months) the participants will be equipped with accelerometers and inclinometers to be worn for 7-days. At session four the participants will receive feedback of their physical activity or sedentary behavior (dependent on which group they are in) based on the assigned intervention. To standardize counselling sessions, coaches will get a checklist with multiple issues that they should address during the session and selected sessions will be recorded. The CBT techniques include 1) Goal setting tied to values and identifying the individual’s resources and boundaries for making behavior changes, 2) Functional analysis including antecedents and consequences of unwanted and wanted behavior, and 3) Acceptance techniques. The active components in the counselling according to the taxonomy of behavior change [20] is described in detail in Additional file 1.

#### Team leaders

The team leaders will play an important role in the delivery of the environmental and organisational components, which is described in detail in Additional file 2. Other important tasks are to encourage employees to participate and remain in the study and to communicate with the research team. Before the start of the study all team leaders will be invited to an information meeting to explain the project and their role in the project. At the start of the intervention all team leaders receive the relevant part (sedentary or physical activity dependent on randomization) of Additional file 2 and will be contacted by phone by one of the researchers to discuss a plan on how to implement the different components, which will be written down and confirmed by email. In case of changes or questions about the defined plans, the team leaders will be asked to contact the research team. After mid-term of the intervention period, the team leaders will be invited to an inspiring lecture and discussion with two experts on how to support behavioral changes. Within two months after the end of the intervention, team leaders will be contacted again by phone to evaluate the intervention. This is also part of a qualitative study planned to evaluate the feasibility of the intervention.

#### Waiting list

The waiting list control will be a passive control group that will be measured again after 6 months. After this measurement they will start the assigned randomized intervention.

### Data collection

Inclusion of the study will start in March 2018 and is planned to end in December 2018. Data of the randomized controlled trial is thus expected to be finished in May 2019 and the two year follow-up by the end of 2020. On all measurement occasions, participants will fill out web questionnaires and perform measurements at an in-house test site at the company. For a complete list of measurements, please see an overview in Additional file 3. This overview includes the specification of which measurements will be performed at which measurement time points.

#### Proximal outcomes: physical activity and sedentary behavior

Participants will be fitted with an Actigraph GT3X accelerometer on the hip during 7 days and on the non-dominant wrist during sleeping-time. Simultaneously, persons will be fitted with an inclinometer (ActivPal3 activity monitors, PAL technologies limited, Glasgow, UK) to measure sedentary behavior. The ActivPal is waterproofed and will be secured to the frontal aspect of the mid-thigh using a 10x10cm adhesive hypo allergic thin plastic film (Tegaderm Roll, 3 M). During the measurement period, participants will be given a diary in which they note sleep and waking times, working hours and any device removals.

#### Actigraph

The GT3X will sample 3-axial acceleration at a sampling frequency of 30 Hz. The accelerometer expresses intensity of movement in counts per minute (cpm). The triaxial acceleration vector magnitude (VM) will be calculated as, where x, y and z denotes the vertical, anteroposterior and mediolateral axes, respectively. Minimum requirement for data inclusion will be 600 min of valid daily monitor wear on at least 4 days. Wear time will be defined by subtracting non-wear time from waken time (as defined from the sleep diary). Non-wear time will be defined as at least 60 consecutive minutes with no movement (VM = 0 counts per minute, cpm), with allowance for maximum 2 min of activity.

Daily physical activity pattern will be presented as 1) percentage of wear time spent in three intensity-specific categories; sedentary, light and moderate-to vigorous physical activity, 2) total volume of physical activity expressed as mean cpm over the study period, 3) time spent in prolonged (> 20 min) periods spent sedentary, 4) number of breaks per sedentary hour, 5) fulfilment of national physical activity recommendations and 6) total physical activity.

#### ActivPAL

This inclinometer registers the inclination of the thigh to distinguish between sitting, standing and walking. The inclinometer will be initialized and processed using the activPAL software, using references from participants’ diaries on waking and working hours. Additional data processing will be performed with the HSC analysis program (developed by Dr. Philippa Dall and Professor Malcolm Granat, School of Health and Life Sciences, Glasgow Caledonian University). Before and after processing, quality controls will be conducted and recorded time will be coded as wear time, non-wear time or working time. Sleep and non-wear time will be excluded. For a day to be considered valid the following rules will apply: 10 ≥ hours of worn waking hours, < 95% of time spent in any one behavior (sedentary, standing, walking) and ≥ 500 steps [23]. Working days will be considered valid when worn for ≥80% of the time at work and 5 ≥ hours of worn working hours. To be included in the analytical sample, data from at least four days is required, with at least two working days and two non-working days. Time spent sitting, standing and walking will be identified for each day and then averaged over the valid days. In addition, sitting bouts will be analysed. Distributions of activities will be reported separately for workplace, non-workplace, working days and non-working days. All results will be presented in hours/day and in % wear time.

#### Distal outcomes: mental health and cognition

##### Mental health

The following self-reported measures will be assessed: Stress [24], Recovery [25], Depression and anxiety [26], Burnout [27], General mental health [28], Well-being [29], Sickness absence [30], Life satisfaction [31], and Performance-based self-esteem [32].

#### Cognition

##### Cognitive test battery

A comprehensive cognitive test battery (11 tests: 7 computerized (E-prime 2.0, Psychology Software Tools Inc.) 4 paper and pen; will be administered (duration: approximately 1 h) [33, 34]. The following cognitive domains will be assessed: *processing speed* (Digit symbol), *attention* (Trail Making Test-A), *working memory* (Capacity: Automated Operation Span; Backward Digit Span), *executive functions* (Trail Making Test-B, Stroop, n-back), *episodic memory* (free recall; recognition), *semantic memory* (SRB:1), and *visuospatial ability* (Form Board Test).

Additionally, subjective memory complaints will be measured using a single item [35].

#### Secondary outcomes

##### Cardiovascular fitness

Participants will undergo a sub maximal cycle ergometer test [36]. Heart rate response to a sub maximal rate of work will be used to estimate maximal oxygen consumption (cardiovascular fitness). Cardiovascular fitness will be expressed as absolute values (liter per minute) and as relative values (mL per minute per liter body mass). Before the test, blood pressure will be determined in sitting position, and a check-list will be used to assess potential contra-indications to participate in this test. In case a contra-indication would be found, such as high blood pressure, the submaximal cycle ergometer test will not be performed.

##### Body composition

Body mass index will be calculated from measured weight and height. Weight will be measured by Tanita BC-418MA Body Fat Analyzer digital scale (Tanita Corporation of America, Inc., Arlington Heights, IL) to the nearest 0.1 kg.

Waist circumference will be measured in duplicate with participants standing dressed in underwear and exhaled, at the minimum circumference between the iliac crest and the rib cage. Measurement will be rounded to the nearest 0.5 cm.

##### Sleep

During night time, participants will wear the accelerometer (Actigraph, as described above) on the non-dominant wrist. Using existing algorithms, the following key aspects of sleep will be calculated: Time to sleep onset, sleep efficiency, times wake up after sleep onset and total sleep time. Additionally, subjects will fill in a sleep diary, by noting time of day for going to bed and waking up and fill in a generic sleepiness questionnaire [37].

#### Self-reported physical activity. Sedentary behavior, and other health habits

- Self-reported data for physical activity, sedentary behavior and active transport will be assessed, using three validated questions [38–40]
- We will assess the use of the physical activity and sedentary behavior strategies, such as the use of an app, exercising during work and walking-and standing meetings
- Other health habits will be assessed, including smoking/snuss, drinking and diet [41, 42]

##### Physical health

Self-reported physical health [43] and self-reported health conditions, e.g. diabetes or intestinal disorders.

#### Working mechanisms

##### Blood analyses

Participants will be instructed to fast for at least eight hours preceding the assessment. Fasting venous blood samples will be obtained from the antecubital vein. Per measurement point, two blood samples (each 5 mL) will be drawn. Cooled (~ 4 °C) samples will be centrifuged to aquire plasma and serum, aliquoted and stored at minus 80 °C. HbA1c will be analyzed using the IFCC method [44]. Plasma and serum levels of inflammatory markers, BDNF [45, 46], VEGF [46], and IGF-1 [46] will be analyzed using enzyme-linked immunosorbent assays following manufacturer’s instructions. Furthermore, genetic profiling of BDNF genes of will be performed [47, 48]. Genomic DNA will be isolated from peripheral white blood cells using the PureGene kit (Gentra Systems, Minneapolis, MN). The Val66Met polymorphism at the BDNF locus will be genotyped using the amplification conditions reported by [49] and detection by fluorescence polarization as described by Chen et al. (1999) [50].

Questionnaires:

- Exercise self-efficacy [51–53], and comparable questionnaire adjusted for sedentary behavior
- For individual barriers [17] and motivation to change a questionnaire was developed based on previous qualitative research [54–56]
- Feasibility and acceptability of interventions (self-developed questionnaire)
- Self-regulation [57]
- Over commitment [58]
- Work/non-work interference and enhancement [59, 60]
- Job Demand-control-support [61]
- Job insecurity [62]
- Work engagement [63]
- Work climate [64]
- Health-promoting leadership [65]

Additionally, a qualitative study will be performed on feasibility and acceptability of the interventions. For quality control of the counselling intervention sessions will be recorded.

#### Co-variables

- Demographic and work-related variables, including organizational changes [66]
- Medication

### Data management and quality assurance

All personnel involved in the trial is trained according to standard operating procedures and the principles of good clinical practice. Each participant was assigned a unique numeric identifier code before their first measurement to enable link-anonymisation of data. All personal data will be stored in encrypted files, and links to personal information are only available to the study co-ordination team. Consent forms and paper acquired data are stored in locked filing cabinets. Paper acquired data (fitness tests and part of cognitive tests) are entered by a research assistant unaware of group allocation. Random checks of the entered data against the source document are performed and outlying values are double checked with the source document. Furthermore, all data are checked on appropriate range and consistency by trained research assistants.

### Sample size

Sample size was calculated in three steps [67]:

i. Sample size based on an individualized randomized controlled trial
ii. Corrected for the design effect, taking into account the clustering
iii. Corrected for unavailable data

The sample size calculation was based on 1 follow-up measurement, with a power of 80% and significance level of 0.05, corrected for 3 groups (0.05/3 = 0.017).

The design effect was 1.77. This was determined, assuming that the proportion of variance accounted for by between cluster variations was 0.05 [68]. Furthermore, we corrected for unequal clusters, assuming an average cluster size of 14 with a standard deviation of 5.83.

Unavailable data were assumed by correcting for the drop out of 3 clusters, with an average cluster size of 14 per group and for unavailability of data (missing, individual drop-outs) for 20% of participants.

For the remaining input of the sample size calculations, data were used from subproject 1 of the larger research project (as described earlier).

Using these input, estimated needed sample sizes are the following:

- Physical activity, moderate to vigorous physical activity, meeting the guidelines

Proportion of participants meeting guidelines in control group: 30%.

Estimated proportion of participants meeting guidelines in intervention groups: 60%.

i. *Sample size based on individualized randomization:* 52 per group
ii. *Corrected for design effect:* 92 per group
iii. *Corrected for unavailable data:* 125 per group

- Sedentary behavior, total sedentary behavior, in hrs/day

Difference in mean value between groups: 1 h/day.

Standard deviation: 1.4 h/day.

i. *Sample size based on individualized randomization:* 41 per group
ii. *Corrected for design effect:* 72 per group
iii. *Corrected for unavailable data:* 101 per group

- Mental health, stress, single item [24]

Proportion of participants with stress complaints in control group: 26%.

Estimated proportion of participants with stress complaints in intervention groups: 5%.

i. *Sample size based on individualized randomization:* 57 per group
ii. *Corrected for design effect:* 100 per group
iii. *Corrected for unavailable data:* 134 per group

- Cognition, stroop test (in seconds)

Difference in mean value between groups: 7 s.

Standard deviation: 9.85 s.

i. *Sample size based on individualized randomization:* 41 per group
ii. *Corrected for design effect:* 73 per group
iii. *Corrected for unavailable data:* 102 per group

This 24- months study includes 5 measurement time points, with measurements every 6 months. The 5th measurement point will only be performed provided that drop-out rate is not higher than 30% and sufficient resources are available [69]. When persons drop-out of the study they will be asked whether they are still willing to wear the accelerometers and/or fill out a short version of the questionnaire including only the main distal outcomes.

### Statistical analyses

In accordance with the research questions, statistical analyses will be conducted to determine whether the groups differ in changes over time in proximal, distal as well as secondary outcomes. Statistical significance will be set at the 5% level (two-tailed). To adjust for company and clustering, analyses will be performed using multilevel modelling. The models will include allocated group (reference group = control group) and baseline values. Both crude models and models adjusted for potential confounders will be determined. Potential confounders will be defined a priori based on the findings from the larger research project. Separate models will be made for the different outcomes regarding physical activity, sedentary behavior, mental health and cognition. All analyses will be performed using both per protocol and intention to treat approaches, were intention to treat is defined as persons attending at least 3 out of 5 counselling sessions.

Moderator analysis will examine whether intervention effects differ across individual (e.g. age, gender and education). Mediator analyses will test whether individual and organizational factors, described under working mechanisms, mediated the intervention effects.

## Discussion

Effective, feasible and sustainable interventions are essential to improve mental health and cognition of office workers. The study is unique in that it is part of a bigger research project “physical activity and healthy brain functions” of which results have been used to design the current study. Furthermore, during the entire research project we are in a continuous dialogue with the involved companies to listen to each other’s ideas and understand choices made. Another strength of this study is the use of multiple objective outcome measures using both accelerometers and inclinometers, taking blood samples, and performing fitness and cognitive tests. This longitudinal study will be an important contribution to the current available evidence of how to improve office worker’s mental health and cognition by targeting physical activity and sedentary behavior.

## Additional files

Additional file 1: Active components of counselling according to the behavior change technique taxonomy (DOCX 15 kb)

Additional file 2: Team leaders’ role in interventions (DOCX 16 kb)

Additional file 3: Overview of measurements (DOCX 16 kb)

## Abbreviations

Cpm: Counts per minute
RCT: Randomized controlled trial
VM: Vector magnitude
