# Supplementary material for: Improving office workers’ mental health and cognition: a 3-arm cluster randomized controlled trial targeting physical activity and sedentary behavior in multi-component interventions
Source: BMC Public Health. 2019 Mar 5;19:266. doi: 10.1186/s12889-019-6589-4 (PMC6402109; doi:10.1186/s12889-019-6589-4)
Supplement: Supplementary file 1 — Active components of counselling according to the behavior change technique taxonomy (DOCX 15 kb) [file 12889_2019_6589_MOESM1_ESM.docx]

**Additional file 1.** Active components of counselling according to the behavior change technique taxonomy

| BCTTv1 categories^¤^ | **Components used in the individualized counselling** | **Components used at the workplace*** |
| --- | --- | --- |
| **1. Goals and planning** |  |  |
| - 1. Goal setting (behavior) | X |  |
| - 1. Problem solving | X |  |
| - 1. Goal setting (outcome) | X |  |
| - 1. Action planning | X |  |
| - 1. Review behavior goal(s) | X |  |
| - 1. Discrepancy between current behavior and goal | X |  |
| - 1. Review outcome goal(s) | X |  |
| 1.9. Commitment | X |  |
| **2. Feedback and monitoring** |  |  |
| 2.2. Feedback on behaviour | X |  |
| 2.3. Self-monitoring of behaviour | X |  |
| **3. Social support** |  |  |
| 3.2. Social support (practical) |  | X |
| 3.3. Social support (emotional) | X |  |
| **4. Shaping knowledge** |  |  |
| 4.1. Instruction on how to perform the behavior | X | X |
| 4.2. Information about Antecedents | X |  |
| 4.3. Re-attribution | X |  |
| **5. Natural consequences** |  |  |
| 5.1. Information about health consequences | X |  |
| 5.3. Information about social and environmental consequences | X |  |
| 5.4. Monitoring of emotional consequences | X |  |
| 5.6. Information about emotional consequences | X |  |
| **6. Comparison of behaviour** |  |  |
| 6.1. Demonstration of the Behaviour | X |  |
| 6.2. Social comparison | X |  |
| **7. Associations** |  |  |
| 7.1. Prompts/cues |  | X |
| 7.2. Cue signalling reward | X |  |
| 7.3. Reduce prompts/cues | X |  |
| 7.4. Remove access to the reward | X |  |
| 7.5. Remove aversive stimulus | X |  |
| 7.6. Satiation | X |  |
| 7.7. Exposure | X |  |
| 7.8. Associative learning | X |  |
| **8. Repetition and substitution** |  |  |
| 8.2. Behavior substitution | X |  |
| 8.3. Habit formation |  | X |
| 8.4. Habit reversal | X |  |
| 8.7. Graded tasks | X |  |
| **9. Comparison of outcomes** |  |  |
| 9.1. Credible source |  | X |
| 9.2. Pros and cons | X |  |
| **10. Reward and threat** |  |  |
| 10.3. Non-specific reward | X |  |
| 10.6. Non-specific incentive | X |  |
| 10.9. Self-reward | X |  |
| **11. Regulation** |  |  |
| 11.2. Reduce negative emotions | X |  |
| **12. Antecedent** |  |  |
| 12.1. Restructuring the physical Environment |  | X |
| 12.2. Restructuring the social environment |  | X |
| 12.3. Avoidance/reducing exposure to cues for the behavior | X |  |
| 12.5. Adding objects to the environment |  | X |
| **13. Identity** |  |  |
| 13.1. Identification of self as role model | X |  |
| 13.2. Framing/reframing | X |  |
| **14. Scheduled consequences** |  |  |
| 14.8. Reward alternative behavior | X |  |
| **15. Self-belief** |  |  |
| 15.3. Focus on past success | X |  |

^¤^Components according to evidence-based Behavior Change Technique Taxonomy v1
* Organisational and environmental components supported by the team leaders, see additional file 2
