# Supplementary material for: Improving office workers’ mental health and cognition: a 3-arm cluster randomized controlled trial targeting physical activity and sedentary behavior in multi-component interventions
Source: BMC Public Health. 2019 Mar 5;19:266. doi: 10.1186/s12889-019-6589-4 (PMC6402109; doi:10.1186/s12889-019-6589-4)
Supplement: Supplementary file 2 — Team leaders’ role in interventions (DOCX 16 kb) [file 12889_2019_6589_MOESM2_ESM.docx]

**Additional file 2.** Team leaders’ role in interventions

| **Intervention to reduce sedentary behavior** | | | **Intervention to promote physical activity** | | |
| --- | --- | --- | --- | --- | --- |
| **Focus** | **What?** | **When?** | **Focus** | **What?** | **When?** |
| Preparations | Preparatory meeting to discuss concrete intervention plans for the action points below | First week of intervention | Preparations | Preparatory meeting to discuss concrete intervention plans for the action points below | First week of intervention |
| Interruption of long-term sedentary meetings | Book meetings in which you motivate or facilitate standing meetings / breaks of sedentary behavior every 20 minutes | At least 3 times / month during the intervention period.  Aim for interruptions every 20 minutes at all meetings | Exercise during working hours | Book one group training session at work time, e.g. during lunch time with the whole cluster or divided into smaller groups when needed | About 3 weeks after the start of the intervention |
| Walking meetings | Encourage employees to have at least 1 walking meeting during the first 2 months | At least once during the first 2 months, preferably every month | Exercise during working hours | Book a group brisk lunch walk with the whole cluster (or divided into smaller groups when needed) | Two times: around week 5 and 15 of the intervention |
| Standing at own desk and during meetings | Encourage interruptions of prolonged sitting during workday (go to printer often, talk to colleagues, low intense physical activity, stand at the desk¤, etc.).  Encourage standing up part of the workday at own desk¤ and during meetings | Throughout the intervention period | Work- and leisure time exercise | Encourage exercise during working hours and leisure, including transport (walking / cycling / jogging) to and from work | Throughout the intervention period |
| Active transportation | Encourage use of provided company cycles. Change passive transportation to meetings into active transportation | Throughout the intervention period | Active transportation | Encourage use of provided company cycles for day time training or active transportation to meetings | Throughout the intervention period |
| Role model | Be a role model by having regular breaks of sedentary behavior, by standing up at part of the workday at your desk and during meetings | Throughout the intervention period | Role model | Be a role model by working out during working days or by using active transportation to and from work | Throughout the intervention period |
| Evaluation | Evaluating meeting with a researcher: discussing the feasibility of the intervention (by skype, 60 min) | Within 2 months after the end of the intervention | Evaluation | Evaluating meeting with a researcher: discussing the feasibility of the intervention (by skype, 60 min) | Within 2 months after the end of the intervention |

¤ The availability of sit-stand desks is common in office-based workplaces in Sweden, although with limited usage (17)
