# Supplementary material for: Improving office workers’ mental health and cognition: a 3-arm cluster randomized controlled trial targeting physical activity and sedentary behavior in multi-component interventions
Source: BMC Public Health. 2019 Mar 5;19:266. doi: 10.1186/s12889-019-6589-4 (PMC6402109; doi:10.1186/s12889-019-6589-4)
Supplement: Supplementary file 3 — Overview of measurements (DOCX 16 kb) [file 12889_2019_6589_MOESM3_ESM.docx]

**Additional file 3. Overview of measurements**

| *Overview of measurements*  Arm 1 and 2  Arm 3: wait list group | **Baseline**  *(0 mths)*  *(-6 mths and 0 mhts)* | **Mid-way**  *(+3 mths)*  *(+3 mths)* | **After intervention**  *(6 mths)*  *(6 mths)* | **Follow-up**  *(12 mths)*  *(12 mths)* | **Long follow-up**  *(18 mths)*  *(18 mths)* | **Long follow-up**  *(24 mths)*  *(NA)* |
| --- | --- | --- | --- | --- | --- | --- |
| **Proximal outcomes** | | | | | | |
| Physical activity (PA): Actigraph | x | x | x | x | x | x |
| Sedentary behavior (SB): ActivPal | x | x | x | x | x | x |
| **End points** | | | | | | |
| Mental health  Self-reported:  - Well-being  - Stress  - Recovery  - Anxiety and depression  - Burnout  - Mental health  - Self-reported sickness absence  - Life satisfaction  - Performance-based self-esteem | x |  | x | x | x | x |
| Cognition  Cognition tests  Subjective memory complaints | x  x |  | x  x | x  x | x  x | x  x |
| **Distal outcomes** | | | | | | |
| Fitness test (including weight + height)  and blood pressure | x |  | x | x | x | x |
| Body composition: BMI + waist circumference | x |  | x | x | x | x |
| Sleep from Actigraph  Self-reported sleep | x |  | x | x | x | x |
| Self-reported PA | x |  | x | x | x | x |
| Self-reported SB | x |  | x | x | x | x |
| Active transport | x |  | x | x | x | x |
| Use of PA and SB intervention strategies | x |  | x | x | x | x |
| Other health habits: smoking/snuss, drinking, diet | x |  | x | x | x | x |
| Physical health  - Self-reported physical health  - Health complaints  - Blood samples | x  x  x |  | x  x  x | x  x | x  x | x  x |
| **Working mechanisms** | | | | | | |
| Biological mechanisms - Blood samples,  including  - Genetic profiling | x  x |  | x |  |  |  |
| Self-efficacy | x | x | x | x | x | x |
| Individual barriers | x |  | x | x | x | x |
| Motivation to change | x | x | x | x | x | x |
| Self-regulation | x | x | x | x | x | x |
| Over commitment | x |  | x | x | x | x |
| Work/non-work interference and enhancement | x |  | x | x | x | x |
| Demand-control | x |  | x | x | x | x |
| Job insecurity | x |  | x | x | x | x |
| Work engagement | x |  | x | x | x | x |
| Work climate | x |  | x | x | x | x |
| Health promoting leadership | x |  | x | x | x | x |
| **Co-variables** | | | | | | |
| Baseline demographic + work | x |  |  |  |  |  |
| Other demographic + work |  |  | x | x | x | x |
| Organisational changes | x |  | x | x | x | x |
| Medication | x |  | x | x | x | x |
